# Genetic worth of multiple sets of cowpea breeding lines destined for advanced yield testing

**DOI:** 10.1007/s10681-020-02763-y

**Published:** 2021-01-29

**Authors:** Patrick Obia Ongom, Christian Fatokun, Abou Togola, Oluwaseye Gideon Oyebode, Mansur Sani Ahmad, Ishaya Daniel Jockson, Garba Bala, Ousmane Boukar

**Affiliations:** 1 International Institute of Tropical Agriculture (IITA), IITA Kano Station, PMB 3112, Kano, Nigeria; 2 International Institute of Tropical Agriculture (IITA), Ibadan, Nigeria

**Keywords:** Cowpea [*vigna unguiculata* (l.) walp.], Genetic advance, Genetic potential, PCA clustering, Usefulness criterion, Multiple populations

## Abstract

The objective of this study was to determine genetic potentials in eight sets of cowpea lines for grain yield (GY), hundred seed weight (HSDWT) and days to 50% flowering (DT50FL). A total of 614 F_6_ genotypes constituting the sets, grouped by maturity, were evaluated across two locations in Northern Nigeria, in an alpha lattice design, two replications each. Data were recorded on GY, HSDWT and DT50FL.Variance components, genotypic coefficient of variation (GCV), and genetic advance (G_A_) were used to decode the magnitude of genetic variance within and among sets. Genetic usefulness (*Up*) which depends on mean and variance to score the genetic merits in historically bi-parental populations was applied to groups of breeding lines with mixed parentage. Principal component analysis (PCA) was used to depict contribution of traits to observed variations. GY and DT50FL explained the variance within and between sets respectively. Genotypes were significantly different, although genotype-by-location and set-by-location interaction effects were also prominent. Genetic variance (δ^2^_G_) and GCV were high for GY in Prelim2 (δ^2^_G_ = 45,897; GCV = 19.58%), HSDWT in Prelim11 (δ^2^_G_ = 7.137; GCV = 17.07%) and DT50F in Prelim5 (δ^2^_G_ = 4.54; GCV = 4.4%). Heritability varied among sets for GY (H = 0.21 to 0.57), HSDWT (H = 0.76 to 0.93) and DT50FL (H = 0.20 to 0.81). G_A_ and percentage G_A_ (G_APM_) were high for GY in Prelim2 (G_APM_ = 24.59%; G_A_ = 269.05Kg/ha), HSDWT in Prelim11 (G_APM_ = 28.54%; G_A_ = 4.47 g), and DT50F in Prelim10 (G_APM_ = 6.49%; G_A_ = 3.01 days). These sets also registered high values of genetic usefulness, suggesting potential application in non-full sib populations. These approaches can be used during preliminary performance tests to reinforce decisions in extracting promising lines and choose among defined groups of lines.

**Supplementary Information:**

The online version contains supplementary material available at (10.1007/s10681-020-02763-y)

## Introduction

Cowpea [*Vigna unguiculata* (L.) Walp.] is a key legume in the semi-arid regions of Sub-Saharan Africa (SSA) because of its significant contribution to food and nutritional security in the region. The crop provides a cheap source of quality protein and minerals to both rural and urban communities in Africa (Ajeigbe et al. 2012; Dube and Fanadzo 2013). The grains and leaves are both good sources of protein ranging from 21 to 33% and from 27 to 43%, respectively (Ahenkora et al. 1998; Boukar et al. 2011; Ddamulira et al. 2015). Cowpea predominance in the dry zones of Africa is attributable to its inherent drought tolerance and capability to grow in marginalized soils where other crops fail (Ehlers and Hall 1997; Ewansiha and Singh 2006; Agbicodo et al. 2009; Hall et al. 2010; Fatokun et al. 2012). In the dry savannas of West Africa, cowpea is regarded as a dual purpose crop providing both human food and animal fodder (Singh et al. 2003; Kamara et al. 2012). Additional attractiveness of cowpea is seen in its ability to fix nitrogen in the soil, making it a key component of the traditional intercropping systems (Kyei-Boahen et al. 2017). A recent report also revealed cowpea’s medicinal properties, particularly anti-cancer, anti-hyperlipidemic, anti-inflammatory and anti-hypertensive properties (Jayathilake et al. 2018). These unique properties make cowpea a focus crop with potential to curb both the dynamic climate and malnutrition challenges in SSA.

Cowpea is largely produced and consumed in west and central Africa, with Nigeria leading the production at a rate of 2.14 million metric tonnes annually (FAOSTAT 2017; Boukar et al. 2018). However, farmers in west Africa have not been able to exploit the crops’ yield potential, given that the average grain yield is about 492 kg/ha compared to a possible yield of between 2,000 and 3,000 kg/ha demonstrated on experimental station (Carsky et al. 2001; Agbicodo et al. 2009; Ahmad et al. 2010; Boukar et al. 2013, 2018). The production and consumption of cowpea is challenged by numerous biotic and abiotic factors including insects, diseases, parasitic weeds, extreme and intermittent water and heat stresses (Agbicodo et al. 2009; Boukar et al. 2013, 2018; Togola et al. 2017).

Concerted efforts are being placed on cowpea to boost its productivity including deployment of modern quantitative genetics and genomic tools (Ehlers et al. 2012; Boukar et al. 2016, 2018). These are expected to accelerate the rate of genetic gain, allowing farmers to benefit from the full genetic potentials of the crop. Additionally, the need to meet consumers’ demand has revolutionized breeding, now requiring breeding for clearly defined product targets and profiles (Ragot et al. 2018). Grain yield, fodder potential and maturity duration are key components of each product target among other traits. Consequently, breeders may have to create and parallelly manage multiple populations of genetic materials in the breeding programs to suit specific product targets. Breeding lines emerging from several crosses may be fragmented based on maturity groups or other traits. In cases where multiple breeding sets are created, it is important to understand the genetic potentials of each set of materials or populations in terms of genetic variability and expected genetic advance for key product traits like grain yield to warrant continued investment in advanced testing across the target environments (Allier et al. 2019). The approach to define the usefulness or the genetic worth of a set of genetic materials or a cross has been described (Bernado 2010; Allier et al. 2019) and the concept has been largely applied in maize breeding to identify the best populations for extraction of superior inbred lines (Tabanao and Bernardo 2005). In this approach, the genetic usefulness (*U*) of a population for a given quantitative trait is determined by its mean (*μ*) and expected genetic gain (*iHσ*_*p*_) as follows: *U* = *μ* + i*H*σ_*p*_ where *i* is the selection intensity which depends on the selection pressure, *σ*_*p*_ is the phenotypic standard deviation, and *H* is the broad sense heritability (Tabanao and Bernardo 2005; Bernado 2010). For instance, mean and genetic variance components of grain yield and other traits were deployed to dissect the usefulness of nine (6 synthetic and 3 F_2_) maize populations (Fountain and Hallauer 1996). In cowpea and soybean, Meenatchi et al. (2019) and Johnson (1955) exploited the genetic variability parameters: phenotypic coefficient of variation (PCV), genotypic coefficient of variation (GCV), broad sense heritability (H) and genetic advance as a percentage of mean (G_APM_) for grain yield and component traits to understand the extent of genetic variability using F_2_ populations, although the usefulness criterion was not used. Two early generation populations of cowpea were examined based on genetic variance, heritability and genetic advance expressed as a percentage of mean to gauge the degree of genetic variability for grain yield and fodder traits (Kumar et al. 2017; Dinakar et al. 2018). However, when dealing with multiple populations, a combination of the means and genetic advance becomes handy to ease decisions in choosing the best sets of materials to advance in the breeding program (Schnell and Utz 1975; Tabanao and Bernardo 2005; Bernado 2010). The use of these genetic parameters is key in predicting the genetic worth of different sets of breeding populations and therefore reinforcing the decisions to focus resources for advanced testing on lines from populations with high genetic value. The objective of the present study was to decode the genetic potential of eight sets of cowpea breeding materials evaluated in preliminary yield trials to ascertain effective extraction of the best lines for further testing in advanced yield trials and/or for recycling as parents in the hybridization nursery. The study exemplified an effective use of quantitative genetic concepts to make selection decisions in a breeding program.

## Materials and methods

### Site description

Field experiments were conducted during the 2019 cropping season in 2 locations at IITA experimental farms in Minjibir, Kano State, Nigeria, and at the National Animal Production Research Institute (NAPRI), Shika, Kaduna State, Nigeria (Table 1). Minjibir (12° 08.997 ′N, 8° 39.733′ E) is in the Sudan savanna agroecology. The area has one wet season which commences in May/June, ending in October, with mean annual rainfall of about 674 mm and annual temperature range of 26–32 °C. Shika (11° 15′N, 7° 32′E) is in the Northern Guinea Savanna agroecology, in the sub-humid zone of Nigeria. The zone has a unimodal wet season which begins in April/May and finishes by mid-October, with average annual rainfall of 1050 mm. Maximum temperature in Shika during the cropping season varied between 27 and 35 °C. Fertilizer was applied in both fields at a rate of 100 kg of NPK (15–15–15) per ha.

**Table 1:** Descriptions of experiements and sites used for evaluation of 614 cowpea lines, grouped into 8 sets, in year 2019 across two location in Northern Nigeria

Set	Size	Design	^a^Rep	Sowing date		^b^An.Temp Range (o^c^)	^c^An.Rainf (mm)
Prelim1	80	8 × 10 Alpha lattice	2	Minjibir: June 29^th^	Shika: Aug 20th		
Prelim2	78	6 × 13 Alpha lattice	2	Minjibir: June 29th	Shika: Aug 20th		
Prelim3	72	8 × 9 Alpha lattice	2	Minjibir: June 30th	Shika: Aug 21st	Minjibir: 26 to 32	Minjibir: 674
Prelim5	80	8 × 10 Alpha lattice	2	Minjibir: June 29th	Shika: Aug 20th		
Prelim7	78	6 × 13 Alpha lattice	2	Minjibir: June 29th	Shika: Aug 21st		
Prelim8	72	8 × 9 Alpha lattice	2	Minjibir: June 29th	Shika: Aug 21st	Sheika: 27 to 35	Sheika: 1050
Prelim10	64	8 × 8 Alpha lattice	2	Minjibir: Aug 2nd	Shika: Aug 21st		
Prelim11	90	9 × 10 Alpha lattice	2	Minjibir: Aug 2nd	Shika: Aug 20th		

^a^number of replications; ^b^mean annual temperature range measured in degrees celcious (^o^c); ^c^ mean annual rainfal measured in millimeters (mm); Minjibir and Shika are the names of loctions or sites

### Plant genetic materials

Sets of lines belonging to eight cowpea populations intended for preliminary yield tests (PYT), derived from multiple crosses in the breeding program and targeting different product profiles were used in this study. The crossing structure, pedigrees and agronomic features of parental lines are presented in Supplemental File 1. The creation of the multiple sets of test lines was based on maturity duration meant to suit different agro-ecolozies in cowpea growing corridors of Northern Nigeria. Consequently, the sets were categorized as: extra early and early maturity targeting the short duration production in the Sahelian and Sudan Savanna zones of West Africa, Medium and late maturity groups meant for the Medium and late duration product profiles suitable for the Guinea Savanna zone of West Africa. These maturity groups in addition to striga resistance status of the lines gave rise to the eight sets used in the present study. Smarmily, the sets were created by making several bi-parental crosses using specific elite parents per maturity group; that is, two sets for short duration group: Prelim7 and 10, two sets of medium duration group: Prelim2 and 5, and three sets of late duration group: Prelim2, 3 and 8. The crosses generated F_1_s that were self-pollinated and between 200 -300 F_2_ derived lines per set were advanced by single seed descent (SSD) until F_5_ generation. At this stage lines were planted in a striga infested observation plot and susceptible lines within each set were dropped and resultant sets of F_6_ genotypes belonging to the different maturity groups were then used in the present study (Supplemental File 1). Included in the study is an extra early duration set of F_6_ lines referred to as Prelim11 that came from the inter-mating of eight parents. The sets had variable population sizes ranging from 60 to 90 and totaling to 614 genotypes (Table 1). Additionally, the crosses producing the eight sets of genetic materials involved parental lines capturing key traits of focus in the breeding program: High grain yield potential, large seed size, varying maturity (extra-early, early, medium and late), striga resistance, bacterial blight resistance and aphid resistance. The populations were developed by the cowpea breeding program over a period at the International Institute of Tropical Agriculture (IITA), Kano Station, Nigeria.

## Experimental layout

At both Minjibir and Shika experimental sites, the eight populations were laid out as separate experiments in one mega experimental field per location. Materials were planted on ridges spaced at 0.75 m apart, with 0.2 m hill spacing within row. All experiments consisted of four rows per plot, each measuring 4 m long, arranged as an alpha lattice design, with two replications per experiment and the number of incomplete blocks within a replication varied depending on the number of lines within each of the eight populations (Table 1). The experiments at both locations were planted at varying dates in between June and August 2019 depending on suitable cropping period of the location (Table 1).

## Data collection

Plant stand was determined two weeks after seedling emergence and at harvest. Date to 50% flowering (DT50FL) was recorded when 50% of plants in the middle two rows in a plot had flowered and the number of days were computed with reference to the planting date. At maturity, the middle two rows in a plot were harvested, threshed and weighed to obtain grain yield (GY) in grams per plot. The grain yield per plot was then converted to kilograms per hectare (kg/ha), considering the spacing and the plot length. Seed samples were taken from each plot and used to generate the one hundred seed weight (HSDWT) data, measured in grams.

## Data analysis

### Traits distribution

The R statistical software, version 3.5.2 (R Core Team 2018) was used to generate and summarize a graphical visualization using box plots and histograms of traits distribution within and between populations. The means from two locations were used to generate the box plots for the sets while the histograms were generated using individual plot data for the two locations. Scripts used have been provided in Supplemental File 2.

### Mean squares

Analyses of variances (ANOVA) were performed in two steps; first with merged data of all sets, across two locations to assess differences between sets, and second for each population independently to assess variances within the sets. The following models were implemented in R using agricolae and lme4 packages (Bates et al. 2015; Mendiburu 2020) to obtain mean squares (MS), coefficient of variations (CV) and standard errors of means for the traits:

- (a) Between set Model $P_{ijkh} = \mu + set_{i} + l_{j} + \left( {set*l} \right)_{{ij{ }}} \, + \,set\left( g \right)_{{ik{ }}} \,+ \,(set\left( g \right)*l )_{{ijk{ }}} + pooled error$ Where ${P}_{ijkh}$ is the observed value of the ith genotype in the jth location, $\mu$ is the general mean,${set}_{i}$,${g}_{i}$,${l}_{j}$,${(g*l)}_{ij}$, ${set(g)}_{ik}$ and $({set\left(g\right)*l )}_{ijk}$ represent the effects of the genotype, location, the interaction between genotype and location, the effect of genotypes nested within sets and the interaction between genotypes within set by location effect respectively. The between sets ANOVA was performed on a cell mean basis and later converted on a plot basis by multiplying the MS by a common factor$n=p/\sum (1/{r}_{i})$, and the pooled error inserted in the ANOVA was estimated from the experimental error mean squares (EMS) of the individual trials as: $\sum (r*EMS)/\sum r$ (Cochran and Cox 1957). In both expressions mentioned above, ${r}_{i}$ is the number of replications in each trial and $p$ is the number of trials. The approximate degree of freedom for the poled error term was obtained following the Welch–Satterthwaite equation: ${(df \approx (\sum {k}_{i}{EMS}_{i})}^{2}/\sum {(({k}_{i}{EMS}_{i})}^{2}/{v}_{i})$ where${k}_{i}=1/{(v}_{i}+1)$, ${v}_{i}$ is the error degree of freedom of individual trials and ${EMS}_{i}$ is the error mean square of individual trials (Satterthwaite 1946). When conducting F-tests, the denominator term for Set was Set*Loc, while $set\left(g\right)*l )$ was used as a denominator term for the following factors: Loc, Set*Loc and Set(Geno). The pooled error MS was used as a denominator F-test for the Set(Geno)*Loc term.
- (b) Within set Model $Ps_{ijkh} = \mu + g_{i} + l_{j} + l\left( r \right)_{jk} + (l\left( {r\left( b \right)} \right)_{jkh} + \left( {g*l} \right)_{{ij{ }}} + e_{ijkh}$ Where ${P}_{ijkh}$ is the observed value of the ith genotype in the jth location, $\mu$ is the general mean, ${g}_{i}$, ${l}_{j}$, ${l(r)}_{jk}, {(l(r\left(b\right))}_{jkh}$ and ${(g*l)}_{ij}$ represent the effects of the genotype, location, replication nested within location, block and replication nested within location, and the interaction between genotype and location respectively;; and ${e}_{ijkh}$ is the residual effect. The denominator F-test for Loc, Loc(Rep) and Loc(Rep (Block) were $l(r)$, $l(r\left(b\right)$ and EMS respectively while lattice effective error (LEE) was used as a denominator test for Geno and Geno*Loc. The LEE was obtained from the standard error of the mean (SEM) estimates of the Geno*Loc term as: $LEE={n*SEM}_{g*l}^{2}$ where $n$ is the number of values used to estimate the Geno*Loc means which is equal to the number of replications in this case. The R scripts used for these analyses are provided in Supplemental File 2.

### Variance components

To obtain variance components within each set, a linear mixed model (*lmer*) function in R was implemented using lme4 package (Bates et al. 2015). Variance components for the major sources of variation were estimated as;

- Error variance$$\left( {\sigma _{e}^{2} } \right){\mkern 1mu}={\mkern 1mu} MS_{e}.$$
- Genotype × location variance component$\left( {\sigma^{2}_{G \times L} } \right)\, = \,(MS_{G \times L} - MS_{e} )/r.$
- Genotypic variance component $(\sigma^{2}_{G} )\, = \,[(MS_{G} {-}r \, \sigma^{2}_{G \times L} )]/(r\, \times \,l).$
- Phenotypic variance $$\left( {\sigma^{2}_{p} } \right)\, = \,\sigma^{2}_{G} \, + \,\left( {\sigma^{2}_{G \times L} } \right)/l\, + \,\left( {\sigma^{2}_{e} } \right)/r*l.$$

Where, *MS*_*G*_*, MS*_*G* × *L*_ and *MS*_*e*_ are the respective mean squares for genotypes, genotype × location interaction and the error, while *r* is the number of replications and *l* is the number of locations.

### Genotypic and phenotypic variability

The extent of dispersion or the degree of variability within each breeding set was estimated using the formula proposed by (Johnson 1955) as;

Genetic coefficient of variation $\left( {GCV} \right)\, = \,[\left( {\sigma^{2}_{G} } \right)/\mu ]\, \times \,100.$

Phenotypic coefficient of variation$\left( {PCV} \right)\, = \,[\left( {\sigma^{2}_{P} } \right)/\mu )]\, \times \,100$; Where; $\mu$ is the grand mean.

Broad sense heritability (H^2^), was computed from the variance components, expressed on an entry mean basis as:

$$H^{2} = \sigma^{2}_{G} /[(\sigma^{2}_{G} + \, (\sigma^{2}_{G \, \times \, L} )/l \, + \, (\sigma^{2}_{e} )/r \, \times \, l]$$

where *σ*^*2*^_*G*_, *σ*^*2*^_*G* × *L*_ and *σ*^*2*^_*e*_ are variance components for genotype, genotype x location interaction and the error respectively while *r* and *l* are number of replications and locations respectively.

### Genetic advance and usefulness

Expected genetic advance (G_A_) and genetic advance expressed as a percentage of the mean (G_APM_) for each trait was computed according to (Allard 1960) as;

$$G_{A} = \, k_{i} x \, H^{2} x\sigma_{P} ; G_{APM} = \, (G_{A} /) \, \times 100$$

where, *k*_*i*_ is a standardized selection differential (assuming 10% selection intensity for prediction of genetic advance, *H*^*2*^ is the broad sense heritability, σ_P_ is the phenotypic standard deviation, and $\mu$ is the grand mean.

The genetic worth or usefulness (*U*) of each population was then estimated based on the mean and genetic advances according to (Schnell and Utz 1975; Tabanao and Bernardo 2005; Bernado 2010) as: *U* = $\mu$  + *G*_*A*_*.*

Where, *U* is the genetic usefulness of a population, $\mu$ is the mean of the population and *G*_*A*_ is the expected genetic advance.

### Principle component analysis (PCA)

Three parameters namely, yield, seed weight and days to 50% flowering were used to conduct PCA on the sets of breeding lines in R using *vqv/ggbiplot* package developed by Vincent (2011). PCA scores of the three variables namely GY, HSDWT and DT50F were generated and used to determine the contribution of each variable to the total variations within and among the sets. PCA plots were then generated to visualize the scatter pattern of sets and genotypes within sets along the X and Y axes.

## Results

### Traits distribution

**Fig. 1:**
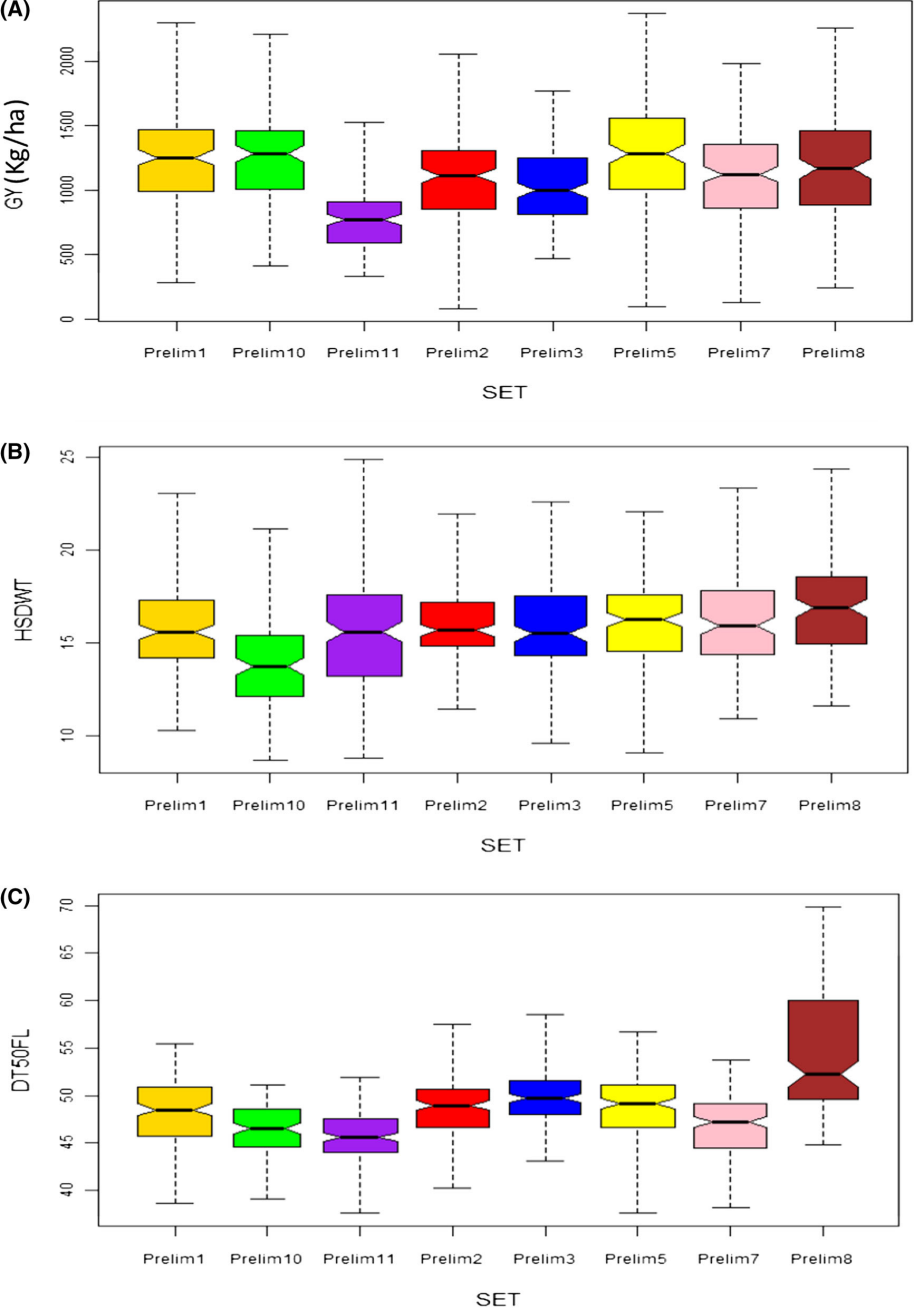
Phenotypic distributions: Box plots showing the dispersion quartiles within each of the eight sets of advanced breeding materials. **a** Grain yield (GY). **b** 100 seed weight (HSDWT). **c** Days to 50% flowering (DT50FL), generated using means from two locations. Histograms reflecting the distributions within each breeding set and boxplots for individual location dispersions are presented in Supplemental Fig. 1

The frequency distributions of lines in each population according to traits are presented in Fig. 1 and Supplemental Fig. 1. The box plots revealed different levels of dispersion within each breeding set with Prelim5 being the most variable set with high median GY, followed by Prelim10 and Prelim1, while Prelim11 had the least dispersion and the lowest median GY (Fig. 1a). The median seed weights (HSDWT) were within close ranges for most of the breeding sets, although Prelim8 stood out with the highest values while Prelim11 had the lowest (Fig. 1b). Prelim11 was earlier than other sets with median DT50FL of about 45 days while Prelim8 took more than 50 days on average to flower (Fig. 1c). The depictions from the histograms showed variations for grain yield, 100 seed weight and days to 50% flowering within the eight sets of breeding materials thus portraying continuous distributions typical of quantitative traits (Supplemental Fig. 1). The eight breeding sets responded uniquely to the environments based on their performances for GY, HSDWT and DT50FL, with each breeding set showing differential performances (high or low) between the two locations as depicted in the individual location boxplots presented in Supplemental Fig. 1.

### Classification of breeding sets

**Fig. 2:**
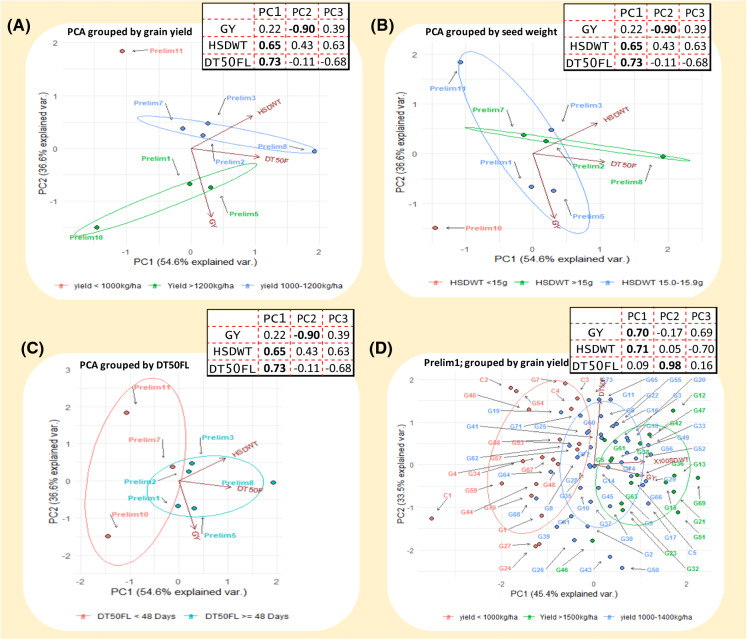
Principal component anaysis showing the diversity between and within eight sets of cowpea lines clustered based on grain yield (GY), 100 seed weight (HSDWT) and days to 50% flowering (DT50FL). **a** PCA cluster grouped by grain yield and highlighting sets with the highest (GY > 1,200 kg/ha), intermediate (1,000 < GY < 1,200 kg/ha) and the lowest (GY < 1,000 kg/ha). **b** PCA cluster grouped by seed weight and highligting sets with the highest (HSDWT > 15 g), intermediate (HSDWT = 15 g) and the lowest (HSDWT < 15 g). **c** PCA cluster grouped by days to 50% flowering and highlighting sets with early (DT50FL < 48 days) and late DT50FL > 48 days) maturing durations. **d** PCA cluster for one of the breeding sets (Prelim1) grouped by grain yield showing diversity within the set and highligting genotypes that are high, intermediate and low yielding within the population. The arrows pointing to the variables (GY, HDSWT and DT50FL) indicate the direction of traits contribution to variation explained by PC1 and PC2. At the top right corner of each PCA plot, are PC scores for the three variables, reflecting the magnitude of contribution of each trait to the variations explained by PC axes. In plots **a, b** and **c** the DT50FL accounted for most of the variation explained by PC1 while GY explained a greater proportion of variance on the PC2 axis. In plot **d**, GY and HSDWT accounted for most of the variance on the PC1 axis while DT50FL was associated with variation on the PC2 axis

Results of PCA conducted among and within breeding sets are presented in Fig. 2 and Supplemental Fig. 2. In general, PCA has showed diversity both within and between breeding sets based on GY, HSDWT and DT50FL, with PC1 and PC2 between sets accounting for 91.2% of total variation in the data. PCA showed the three traits (GY, HSDWT and DT50FL) to be distinct enough and provided good discrimination among and within the breeding sets. For variation among sets, PC1 was strongly associated with HSDWT (PC1 = 0.65) and DT50FL (PC1 = 0.73) and therefore, Prelim sets with high positive scores for PC1 were promising for these two traits, while PC2 was correlated with GY (PC2 = -0.90) hence, sets with high negative scores for PC2 were good for GY. When the data was grouped sequentially by each trait, clusters of breeding sets with potential for GY, HSDWT and DT50FL became apparent. Prelim1, 5 and 10 clustered in a group with GY above 1,200 kg/ha while Prelim11 was alone in the low yielding category (< 1000 kg/ha), the rest being intermediate (Fig. 2a). For HSDWT, Prelim7, 2, and 8 were categorized as having seed weights above 15.9 g, other sets being between 15.0 and 15.9 g, while Prelim10 had a mean HSDWT of less than 15 g (Fig. 2b). PCA for DT50FL revealed two groups with Prelim7,10 and 11 falling in the early flowering category with less than 48 days while the rest of the sets were categorized as flowering later than 48 days after planting (Fig. 2c).

**Fig. 3:**
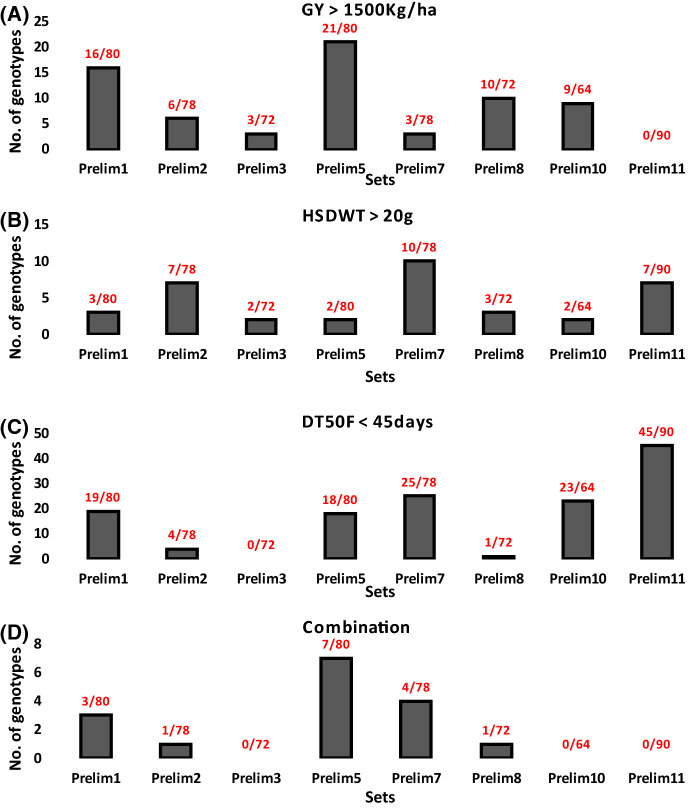
Proportion of best performing genotypes within breeding sets. Bar chats show the number of genotypes out of the population size of each set with; **a** Grain yield (GY) above 1,500 kg/ha. **b** Hundred seed weight (HSDWT) above 20 g. **c** Days to 50% flowering (DT50F) less than 45 days. **d** combination of the three traits (GY ≥ 1,200 kg/ha, HSDWT ≥ 15 g and DT50FL ≤ 45 days)

When we performed PCA within each of the eight sets, it was clear that potentially high yielding genotypes (> 1500 k/ha) that overlap with high seed weight and earliness could be extracted from these populations (Fig. 2d and Supplemental Fig. 2). Except for Prelim7 and 10, GY was strongly correlated with PC1 and accounted for most of the variation among genotypes within sets, therefore, genotypes with high positive scores on PC1 axis were good for GY. For Prelim7 and 10, HSDWT and DT50FL contributed most to the variations explained by PC1. The PCA results further revealed that despite the genotypes being clustered as the best performers for a particular trait, there were still enough diversity among genotypes within each cluster (Fig. 2d and Supplemental Fig. 2). A summary chart for the proportion of best genotypes that could be extracted from each set for GY, HSDWT and DT50F is presented in Fig. 3. It was evident that no genotype with GY above 1500 kg/ha could be obtained from Prelim11 while Prelim5 had the highest number of high yielding genotypes (Fig. 3a). For HSDWT, Prelim7 had a higher number of genotypes with seed weight above 20 g compared to other breeding sets (Fig. 3b). Genotypes with less than 45 DT50F were frequent in Prelim11 while all the genotypes in Prelim3 flowered later than 45 days (Fig. 3c). When the three traits were considered together, more genotypes combining GY > 1,200 kg/ha, HSDWT > 15 g and DT50FL < 45 days could be extracted from Prelim 5 than in other sets (Fig. 3d).

### Variance between and within breeding sets

Analysis of variance depicted the eight breeding sets not to be statistically different for GY, HSDWT and DT50FL, indicated by non-significant mean square values for sets (Table 2). However, numerically, Prelim5 and 8 had higher GY and HSDWT respectively than others. The mean values showing numerical performance of the breeding sets in each of the two locations have been presented in Supplemental Table 1. When we considered genotypes nested within sets, the genotypic differences were highly significant (*P* < 0.001) for all the three traits (Table 2). In addition, the effect of location was highly significant for all the three traits (*P* < 0.001) and consequently, the overall responses of the breeding sets were highly influenced by environment as portrayed by significant Set-by-Location interaction effects for all the three traits (Table 2). The effects of genotypes nested within breeding sets were also statistically significant at *P* < 0.001) for all the three traits, signaling the apparent difference among genotypes within the sets (Table 2). However, the interaction between nested effect of genotype within set and location was highly significant suggesting the presence of genotype-by-environment interaction**.**

**Table 2:** Mean squares among eight sets of advanced breeding materials for grain yield (GY), hundred seed weight (HSDWT) and days to 50% flowering (DT50FL), evaluated across two locations in Northern Nigeria during the rainy season of year 2019

	GY		HSDWT		DT50FL		^#^Denominator F-test
SOURCE	DF	MS	DF	MS	DF	MS	
Set	7	7543948^ ns^	7	179.92^ ns^	7	179.92^ ns^	Set*Loc
Loc	1	2,365,698***	1	689.32***	1	689.32***	Set(Geno)*Loc
Set*Loc	7	4,654,466***	7	121.24***	7	121.24***	Set(Geno)*Loc
Set(Geno)	596	293,674***	595	22.08***	596	22.08***	Set(Geno)*Loc
Set(Geno)*Loc	584	158,036***	595	2.98***	596	2.98***	Pooled error
Pooled error	831	67,023	942	1.93	841	4.30	
Grand mean		1118.57		15.77		48.52	
SEM		129.44		0.69		1.04	
CV%		23.14		8.80		4.27	

*Geno* Genotype; *Loc* Location; *SEM* Standard error of the mean; *CV* Coefficient of variation; *DF* Degrees of freedom; *MS* Mean square; *GY* Grain yield; *HSDWT* Hundred seed weight; *DT50FL* Days to 50% flowering. ANOVA was conducted using genotype means obtained from individual location analysis. The symbols; *, **, and *** represents the probability at 0.05, 0.01 and 0.001, respectively. ^#^ Refers to the source of variation whose degree of freedom was used as denominator for the F-test

When mean squares for variation within breeding sets were compared, significant genotype effects were observed for GY, HSDWT and DT50F with at least a probability value of *P* ≤ 0.05 for all the eight breeding sets (Table 3 and Supplemental Table 2). Location effects were significant, with at least *P* ≤ 0.05 for most of the traits with exceptions in some breeding sets which shown no statistical significance (Supplemental Table 2). The interactions between genotypes and location were also highly significant in all the eight breeding sets for all the three traits (Table 3 and Supplemental Table 2).

**Table 3:** Mean squares within eight sets of advanced breeding materials for grain yield (GY), hundred seed weight (HSDWT) and days to 50% flowering (DT50FL), evaluated across two locations in Northern Nigeria during the rainy season of year 2019

	Traits	Geno	Geno*Loc	EMS	LEE	Mean	Min	Max	SEM	CV%
Prelim1										
DF		79	79	130	130					
MS	GY	213,765***	135,067***	48,836	36,234	1,2378	514	1,819	135	15.4
	HSDWT	20.8***	2.71***	2.2	1.2	15.7	7.3	21.4	0.8	7.0
	DT50F	31.1***	13.1***	4.4	3.3	47.9	41.3	52.5	1.3	3.8
Prelim2										
DF		77	77	134	134					
MS	GY	213,744***	89,578***	53,071	31,752	1,094	1667	1,791	126	16.3
	HSDWT	24.3***	1.88***	1.6	0.8	16.0	6.5	22.4	0.6	5.6
	DT50F	22.0***	7.7***	4.6	2.7	48.8	41.8	53.8	1.2	3.4
Prelim3										
DF		71	71	114	114					
MS	GY	143,356***	109,007***	55,156	33,748	1,027.0	553	1,611	129.9	17.8
	HSDWT	18.2***	2.8***	1.2	0.9	15.9	12.0	22.0	0.7	5.9
	DT50F	11.9***	15.2***	2.9	2.5	49.9	46.3	54.0	1.1	3.2
Prelim5										
DF		79	79	130	130					
MS	GY	397,585***	292,284***	97,108	71,972	1,271	128	2,028	189.7	21.1
	HSDWT	20.3***	4.3***	3.1	1.8	15.9	8.3	21.3	0.9	8.4
	DT50F	36.3***	18.7***	5.7	4.5	48.5	41.8	58.3	1.5	4.4
Prelim7										
DF		77	77	134	134					
MS	GY	309,134***	155,992***	61,853	45,481	1,098	338	1,862	150.8	19.4
	HSDWT	23.1***	3.4***	2.1	1.4	16.2	7.1	21.3	0.8	7.1
	DT50F	17.4***	12.4***	4.5	3.1	46.8	41.3	52.0	1.3	3.8
Prelim8										
DF		71	71	114	114					
MS	GY	286,337***	179,715***	101,088	55,511	1,179	369	1,695	166.6	19.9
	HSDWT	17.0***	3.70***	1.9	1.4	16.8	13.7	24.0	0.8	7.0
	DT50F	40.0***	32.00***	7.0	5.6	54.2	45.0	60.5	1.7	4.4
Prelim10										
DF		63	63	98	98					
MS	GY	167,201***	115,089***	73,121	27,707	1,237	798	1,715	117.7	13.5
	HSDWT	21.3***	2.6***	1.1	0.9	13.9	9.5	22.1	0.7	6.9
	DT50F	18.1***	3.3***	3.5	1.4	46.5	41.5	50.0	0.8	2.6
Prelim11										
DF		89	89	146	146					
MS	GY	184,667***	126,074***	45,953	32,513	804	418	1,404	127.5	22.4
	HSDWT	31.2***	3.4***	2.2	1.4	15.7	9.9	23.4	0.8	7.6
	DT50F	14.6***	4.6***	1.8	1.4	45.5	39.8	51.0	0.8	2.5

*Geno* Genotype; *Loc* Location; *Rep* Replication; *EMS* Error mean square; *LEE* Lattice effective error; *Min* Minimum; *Max* Maximum; *SEM* Standard error of the mean; *CV* Coefficient of variation; *Df* Degrees of freedom; *MS* Mean square; *GY* Grain yield; *HSDWT* Hundred seed weight; *DT50F* Days to 50% flowering; the symbols; *, **, and *** represents the probability at 0.05,0.01 and 0.001 respectively

### Partitioning of variance within breeding sets

When variance components were computed for GY, Prelim2 (*δ*^*2*^_G*L_ = 25,658; δ^2^_G_ = 45,897) and Prelim10 (*δ*^*2*^_G*L_ = 6,231; δ^2^_G_ = 15,848) displayed low magnitude of variance due to genotype-by-Location interaction relative to the genetic variance component (Table 4). Consequently, Prelim2 recorded the highest genotypic coefficient of variations (GCV = 19.58%) for GY, followed by Prelim7 (GCV = 17.94%), Prelim5 (GCV = 17.38%), Prelim8 (GCV = 17.36%) and Prelim1 (GCV = 14.37%). However, Prelim; 3, 10 and 11 had low genotypic and phenotypic coefficient of variations (Table 4).

**Table 4:** Variance component estimates and proportion of genetic and phenotypic variability for grain yield (GY), 100 seed weight (HSDWT) and days to 50% flowering (D50FL) within eight sets of advanced breeding materials evaluated in 2019 at two locations in Northern Nigeria

Traits	Parameter	Prelim1	Prelim2	Prelim3	Prelim5	Prelim7	Prelim8	Prelim10	Prelim11
GY	µ	1238	1094	1027	1271	1099	1179	1237	804
	δ^2^_G_	31,622	45,897	11,883	48,798	38,835	41,848	15,848	6849
	δ^2^_G*L_	78,461	25,658	41,734	85,533	49,826	42,487	6231	19,585
	δ^2^_e_	50,568	57,831	68,026	101,064	69,665	115,114	81,641	52,634
	δ^2^_P_	83,495	73,184	49,757	116,831	81,164	91,870	39,374	29,800
	GCV(%)	14.37	19.58	10.61	17.38	17.94	17.36	10.18	10.29
	PCV(%)	23.35	24.72	21.72	26.89	25.93	25.72	16.04	21.46
HSDWT	µ	15.72	16.04	15.86	15.98	16.19	16.8	13.95	15.65
	δ^2^_G_	4.43	5.57	4.02	4.11	4.94	3.04	4.57	7.137
	δ^2^_G*L_	0.36	0.07	0.57	0.70	0.63	0.66	0.60	0.39
	δ^2^_e_	2.05	1.61	1.24	3.00	2.06	2.42	1.38	2.30
	δ^2^_P_	5.13	6.01	4.62	5.21	5.77	3.97	5.22	7.91
	GCV(%)	13.39	14.72	12.64	12.69	13.73	10.38	15.32	17.07
	PCV(%)	14.40	15.29	13.54	14.29	14.84	11.87	16.37	17.97
DT50F	µ	47.96	48.76	49.99	48.53	46.76	54.23	46.45	45.52
	δ^2^_G_	4.52	2.31	4.40	4.54	1.02	2.07	3.6	2.38
	δ^2^_G*L_	3.79	5.52	2.16	6.56	3.97	12.55	0.02	1.53
	δ^2^_e_	4.80	5.08	4.50	5.70	4.60	7.00	3.30	1.36
	δ^2^_P_	7.62	5.21	6.34	9.24	4.16	10.09	4.43	3.49
	GCV(%)	4.43	3.67	4.20	4.39	2.16	2.65	4.08	3.39
	PCV(%)	5.75	4.68	5.04	6.27	4.36	5.86	4.53	4.10

*µ* overall mean of the breeding set; *δ*^*2*^_*G*_ Genetic variance component; *δ*^*2*^_G*L_ Genotype-by-Location variance component; *δ*^*2*^_*e*_ Error variance component; *δ*^*2*^_*P*_ Phenotypic variance; *GCV* Genetic coefficient of variation; *PCV* Phenotypic coefficient of variation; *GY* Grain yield; *HSDWT* Hundred seed weight; *DT50FL* Days to 50% flowering; the labels: Prelim1, Prelim2, Prelim3, Prelim5, Prelim7, Prelim8, Prelim10 and Prelim11 are the breeding sets or populations

For 100 seed weight, all the breeding sets showed a generally lower magnitudes of variance attributed to Genotype-by-Location interaction (*δ*^*2*^_G*L_) relative to the respective genotypic variances (δ^2^_G_), with Prelim 11 (δ^2^_G_ = 7.137 vs *δ*^*2*^_G*L_ = 0.39) having the highest genetic variance component (Table 4). Breeding sets with high genotypic variability for seed weight included Prelim11 (GCV = 16.33%; PCV = 17.97%), Prelim10 (CGV = 15.32%; PCV = 16.37%), and Prelim2 (GCV = 14.72%; PCV = 14.72%). The rest of the breeding sets were intermediate while Prelim8 had the lowest variability for seed weight with GCV and PCV of 10.38% and 11.87% respectively (Table 4).

The partitioning of variance within sets for DT50FL revealed variable magnitudes of variances attributed to genetic components, with Prelim1 (δ^2^_G_ = 4.52 vs *δ*^*2*^_G*L_ = 3.79) and Prelim5 (δ^2^_G_ = 4.54 vs *δ*^*2*^_G*L_ = 6.56) having high values of genetic variance components relative to the Genotype-by-Location interaction terms (Table 4). Breeding sets that showed high genotypic and phenotypic variability for days to 50% flowering included Prelim1 (GCV = 4.43%; PCV = 5.75%), Prelim5 (GCV = 4.39%; PCV = 6.27%), Prelim3 (GCV = 4.20%; PCV = 5.04%) and Prelim10 (GCV = 4.08%; PCV = 4.53%). Breeding set with the lowest genotypic variability for HSDWT was Prelim7 which had a GCV of 2.16% (Table 4). For all the traits and breeding sets, the differences between the two parameters (PCV and GCV) were minimal, yet by judging from the standard deviation of genetic variance components, these differences are significant.

### Genetic advance and usefulness of breeding sets

Results for expected genetic advance within the eight breeding sets computed based on broad sense heritability and assuming 10% selection intensity are present in Table 5. Heritability for grain yield computed on an entry mean basis ranged from 0.21 for Prelim3 to 0.57 for Prelim2. Overall, the expected genetic advance (G_A_) for GY was more dependent on heritability than on genetic variance. When G_A_ for grain yield was expressed as a percentage of population means (G_APM_), Prelim2 emerged with the highest percentage of expected genetic advance (G_APM_ = 24.59%; G_A_ = 269.05Kg/ha). This was followed by Prelim7 (G_APM_ = 21.84%; G_A_ = 239.91Kg/ha) and Prelim5 (G_APM_ = 19.77%; G_A_ = 251.27Kg/ha). Other intermediate breeding sets were Prelim8 (G_APM_ = 17.91%; G_A_ = 211.12Kg/ha) and Prelim1 with G_APM_ and G_A_ of 14.21% and 175.83Kg/ha respectively while Prelim3 had the lowest G_APM_ (Table 5).

**Table 5:** Predicted genetic advance and genetic usefulness of eight sets of advanced breeding materials evaluated in 2019 at two locations based on grain yield (GY), hundred seed weight (HSDWT) and days to 50% flowering (DT50F)

Traits	Parameter	Prelim1	Prelim2	Prelim3	Prelim5	Prelim7	Prelim8	Prelim10	Prelim11
GY	µ	1238	1094	1027	1271	1099	1179	1237	804
	H^2^	0.35	0.57	0.21	0.42	0.48	0.40	0.40	0.23
	G_A_	175.83	269.05	83.93	251.27	239.91	211.12	140.57	69.83
	G_APM_ (%)	14.21	24.59	8.17	19.77	21.84	17.91	11.36	8.68
	Usefulness (*Up*)	1413.54	1363.27	1110.93	1522.33	1338.48	1389.75	1377.63	874.13
HSDWT	µ	15.72	16.04	15.86	15.98	16.19	16.8	13.95	15.65
	H^2^	0.86	0.93	0.87	0.79	0.86	0.76	0.80	0.90
	G_A_	3.45	4.00	3.29	3.17	3.62	2.68	3.23	4.47
	G_APM_ (%)	21.92	24.94	20.77	19.84	22.35	15.97	23.12	28.54
	Usefulness (*Up*)	19.17	20.04	19.16	19.16	19.81	19.48	17.18	20.12
DT50F	µ	47.96	48.76	49.99	48.53	46.76	54.23	46.45	45.52
	H^2^	0.59	0.61	0.67	0.49	0.25	0.20	0.81	0.59
	G_A_	2.88	2.46	1.60	2.63	0.88	1.14	3.01	1.92
	G_APM_ (%)	6.01	5.05	3.19	5.41	1.89	2.11	6.49	4.22
	Usefulness (*Up*)	45.08	46.29	48.39	45.90	45.87	53.09	43.44	43.59

*µ* overall mean of the breeding set; *H*^*2*^ Broad sense heritability; *G*_*A*_ Genetic advance; *G*_*APM*_ Genetic advance expressed as a percentage of mean; *Up* Usefulness of a population computed as *Up* µ + G_A_; *GY* Grain yield; *HSDWT* Hundred seed weight; *DT50F* Days to 50% flowering

Consequently, when usefulness criterion was used to compare breeding sets based on grain yield, most of the sets that had high percentage of genetic advance also recorded high usefulness (*Up*) values (Table 5). For instance, Prelim5 had the highest *Up* of 1522.33 kg/ha, flowed by Prelim1 (*Up* = 1413.54 kg/ha), while Prelim11 (*Up* = 874.13Kg/ha) and Prelim3 (*Up* = 1110.93Kg/ha) were the least useful sets for grain yield (Table 5). For HSDWT, the heritability values were relatively high across all breeding sets in the range of 0.76 for Prelim8 to 0.93 for Prelim2 (Table 5). It was observed that breeding sets with high heritability values also showed relatively high prediction values of genetic advance, the best sets being Prelim11 (G_APM_ = 28.54%; G_A_ = 4.47 g) and Prelim2 (G_APM_ = 24.94%; G_A_ = 4.00 g), while Prelim8 (G_APM_ = 15.97%; 2.68 g) registered the lowest percentage value of expected genetic advance (Table 5). Usefulness criterion revealed Prelim11 and Prelim2 as the most useful breeding sets for HSDWT with *Up* values of 20.12 g and 20.04 g respectively, while prelim10 had the lowest usefulness value even though it had moderate percentage value of expected genetic advance. When it came to DT50F, the heritability values were variable between the breeding sets ranging from low (0.20 for Prelim8) to intermediate (0.49 for Prelim5) and high (0.81 for Prelim10). Consequently, breeding sets that showed high predicted genetic advance were Prelim10 (G_APM_ = 6.49%; G_A_ = 3.01 days), Prelim1 (G_APM_ = 6.01%; G_A_ = 2.88 days) and Prelim5 (G_AP_ = 5.41%; G_A_ = 2.63 days). Prelim2 and Prelim11 had intermediate proportion of expected genetic advance while Prelim8 and 3 had the lowest prediction of genetic advance for DT50F (Table 5). To make sense of usefulness criterion for DT50F, the expected genetic advance was deducted from the mean DT50F, thereby, revealing Prelim10 (*Up* = 43.44 days), Prelim11 (*Up* = 43.59 days) and Prelim1 (*Up* = 45.08 days) with high genetic potential for early flowering (Table 5).

## Discussion

Decisions in plant breeding are continuously becoming more complex given the dynamic consumer demands and preferences, and the current issues of climate change. As the human population continues to surge, breeders are constantly under pressure to release improved varieties with high yields and other preferred traits. Consequently, a typical active breeding program often handles multiple populations intended for varied purposes or product targets (Witcombe and Virk 2001). This introduces complex deliberations and challenges relating to handling large sizes of genetic materials, resource allocations and selection decisions at every breeding stage (Witcombe and Virk 2001; Sun et al. 2011). Therefore, Sun et al. (2011) noted that, careful choice of genotypes at each step in a breeding program is key in determining the ultimate success in the next selection stages for genetic advancement. The present study elucidated the genetic worth of eight sets of cowpea breeding materials evaluated in preliminary yield trials across two locations in Northern Nigeria, deploying the concepts of genetic variance, heritability, genetic advance and usefulness criterion to aid in making selection decisions for advancement of materials.

We began by examining the distributions of the three traits; GY, HSDWT and DT50FL, within each of the eight breeding sets. The traits variation approximated continuous distributions within the sets, suggestive of quantitative inheritance. Sinnott (1937) argued that, when phenotypic variation is presumably environmental and or conditioned by multiple genes with minor effects, the distribution is essentially symmetrical. In cowpea, grain yield, seed weight and flowering time are complex traits that exhibits quantitative variations in nature (Lopes et al. 2003; Ishiyaku et al. 2005; Boukar et al. 2016). The present study depicted different levels of total dispersions within the breeding sets, with some sets such as Prelim5, Prelim8 and Prelim11 showing slight shifts towards high GY, HSDWT and less DT50FL respectively. The observed dispersions suggested involvement of genetic factors governing the traits tested and that recovering promising lines from these sets is highly probable.

When the breeding sets were analyzed using PCA, it became apparent that the eight breeding sets were distinct from each other although some of them overlap for the three traits. Grouping the breeding sets by their means in respect to the traits allowed the PCA to highlight the potential sets for GY, HSDWT and DT50FL (Fig. 3). When PCA was examined within each breeding set, the structure reflected diversity among genotypes, but some genotypes were highly associated with GY reflecting their yield potentials while others were more correlated with HSDWT and DT50FL, implying those genotypes performed well for the traits in question (Supplementary Fig. 2). PCA was able to identify the top performing genotypes within each breeding set for the three traits, with clear categorizations of those having GY above 1500 kg/ha, seed HSDWT above 20 g and DT50FL less than 45 days.

A summary of the proportion of high performing genotypes that could be extracted from each breeding set was derived from the PCA and presented in Fig. 3. This chart portrayed Prelim5, Prelim7 and Prelim11 as sets having high frequencies of genotypes with GY above1500kg/ha, seed HSDWT above 20 g and DT50F less than 45 days, while Prelim5 had the highest number of genotypes with good combination of desired values of the three traits. PCA is a powerful data reduction tool that has been used in cowpea conventional breeding for morphological characterization and defining key determinants of grain yield (Oladejo et al. 2016). A study by (Vural and Karasu 2007) deployed PCA using multiple yield component traits to understand which of the factors explained most of the total variance in the data, and found seed weight and pod size to contribute most of the variations. In the present study, the traits distributions and PCA provided an overall picture of total variability and structure in the data among and within the breeding sets. Differences among sets were mostly explained by DT50FL as indicated by higher PC1 score for this trait than others. This observation is consistent with the fact that the sets were created based on maturity and therefore, it is expected that the groups would be distinct in terms of DT50FL. On the other hand, variation among genotypes within sets were mostly explained by GY and HSDWT as reflected by high PC1 scores for these traits. Given the information on the contributions of the traits to variation on the PC1 and PC2 axes it was possible to identify promising sets and genotypes within sets for higher GY. The fact that variability among genotypes within each set was mostly explained by GY and HSDWT implies that selection within the sets for these two traits would be more beneficial than for DT50FL. However, since the phenotypic variability generally was only slightly greater than the genetic variability in these traits, the total dispersion does not reflect wholly the magnitude of genetic variance since it is a combination of genetic and environmental variations and hence, an accurate assessment would require partitioning of total variance into its different components (Bernado 2010).

To unravel the variability between and within the sets, we conducted a two-step classical ANOVA, first between the sets and then for individual breeding sets. Sets did not show significant mean differences for all the three traits considered although numerically some sets had higher mean values than others. However, the effect of genotypes nested within location was significant, an indication that sets are likely different, but its significance could have been masked by environment. Indeed, the analysis revealed significant interactions between sets and location and that of genotypes nested in set by location. This outcome suggested that meaningful selections among sets and genotypes within sets would require testing the materials in multiple locations to eliminate the confounding effect of the environment. In addition, it’s important to understand the amount of variation within the population in addition to the mean in order to make a more informed selection decision (Tabanao and Bernardo 2005; Bernado 2010). The present study tested genotypic variation in the eight sets and found the genotype effects within each set to be significantly different for all the traits except for GY in Prelim11. This suggested that there was enough genetic variability within the sets to warrant selection and recovery of good performing lines. However, the observed significant effects of genotype-by-location interaction for traits in most of the sets suggested presence of variation in relative performance of genotypes between the locations, creating an alert to proceed with caution when merging means from the two locations to make selection within the sets (Mohammadi et al. 2015). Genotypic variation for grain yield, seed weight and flowering time in cowpea are known to be influenced by environments (Adewale et al. 2010; Odeseye et al. 2018). This complicates the selection of superior genotypes, thereby reducing genetic progress (Allard and Bradshaw 1964; Mohammadi et al. 2015). In the present case, decision would be made based on two locations data, and considering that further testing in more locations is expected, selection based on means and with a relatively relaxed selection intensity would be suggested to avoid elimination of potentially stable genotypes for GY at this stage**.**

To further decode the genetic potential of the eight breeding sets, total variance within each set was partitioned to reflect variances attributed to genotype, location and the interaction thereof (Table 4). This allowed further dissection of the breeding sets in terms of genetic coefficient variability, heritability, genetic advance and overall genetic usefulness of the sets. Breeding sets that had high relative magnitude of genetic variance had moderate to high heritability and further depicted relatively high expected genetic advance and genetic usefulness This observation suggested that the sets with high values of genetic variance, genotypic coefficient of variation, heritability, expected genetic advance and genetic usefulness for the traits in question, would respond well to future selection, and superior lines for the traits are extractable from these sets. This finding was consistent with the past studies in cowpea which used similar genetic parameters to evaluate the effectiveness of population response to selection (Damarany 1994; Omoigui et al. 2006; Manggoel 2012; Nwosu et al. 2013). The observed minimal differences between GCV and PCV for all the traits studied implied that the traits are mostly governed by genetic factors with little role of environment in the phenotypic expression of these characters (Manggoel 2012). Therefore, selection for these traits based on phenotypic value may be effective. Manggoel (2012) alluded to the fact that heritability estimates coupled with genetic advance are useful in predicting the resultant effect for the selection of the best individuals from a population. Moderate to high broad sense heritability values observed in the present study suggested that selection within each Prelim set for GY, HSDWT and DT50FL would be beneficial, given the moderate magnitude of environmental influence. The results of usefulness criteria were consistent with that from variance components and genetic advance. This suggested that the concept of genetic usefulness may be used to evaluate the genetic merit of specifically defined groups of breeding materials that are not necessarily derived from a two-parent cross. Usefulness criteria have historically been applied to bi-parental populations with full sib progeny to predict population performance in early generations (Tabanao and Bernardo 2005; Bernado 2010; Allier et al. 2019). The advantage of genetic usefulness is that is captures the overall value of a population in terms of its mean performance and total variance (Tabanao and Bernardo 2005; Bernado 2010; Allier et al. 2019). With homozygous lines and the opportunity for replicated testing at later generations as it is the case in the present study, there is improved prediction accuracy of genetic usefulness. The information may still be helpful at early performance testing phase, especially when there is need to prioritize among several groups of breeding materials. Indeed, our study has demonstrated that there are some sets like Prelim11 (*U*_*P*_ = 874 kg/ha) and Prelim3 (*U*_*P*_ = 1110 kg/ha) with relatively low GY scores that would be dropped at this stage and lines taken back in the crossing nursery for yield improvement.

The present study elucidated the structure and properties of eight sets of cowpea breeding materials that are destined for further testing, revealing the uniqueness of each set and the magnitude of expected gain from selection within each set and the genetic usefulness of each set. The variance component analysis allowed estimation of genetic and phenotypic coefficient of variation, heritability and expected genetic advance. These parameters exposed the genetic potential of eight sets of cowpea breeding lines for GY, HSDWT and DT50FL, revealing sets with high genetic variance and from which superior lines could be extracted to recommend for advanced testing. Estimates of genetic usefulness were generally consistent with results from variance components which provided additional layer of information on the score for genetic merits of the sets. The current study highlights a novel application of usefulness criteria in non-biparental populations with populations defined based on maturity groups. However, comparisons of performance among populations may be limited by the nature of traits used for grouping as in the present case where maturity may be correlated with other traits used for assessing performance. Principal component analysis depicted the relative contributions of the three traits to the variability between and within sets, revealing that more benefit would be obtained by selecting among genotypes within sets based on GY and HSDWT than on DT50FL. These approaches generated relevant information required in making decision for advancement in a conventional breeding program.

## Supplementary Information

Below is the link to the electronic supplementary material.

Supplementary file1 (xlsx 37 kb)

Supplementary file2 (DOCX 31 kb)

Supplementary file3 (DOC 1,937 kb)

Supplementary file4 (DOCX 423 kb)

Supplementary file5 (txt 3 kb)

Supplementary file6 (DOCX 16 kb)

## Data Availability

Data generated during this study are included in this published article and its supplemental files. Requests for additional information regarding the elite genetic materials in this study can be made to the authors and will be considered without undue reservation.
