# Supplementary material for: Genetic worth of multiple sets of cowpea breeding lines destined for advanced yield testing
Source: Euphytica. 2021 Jan 29;217(2):30. doi: 10.1007/s10681-020-02763-y (PMC7846544; doi:10.1007/s10681-020-02763-y)
Supplement: Supplementary file 2 — Supplementary file2 (DOCX 31 kb) [file 10681_2020_2763_MOESM2_ESM.docx]

**Supplementary Table 2** Mean squares within breeding sets for grain yield (GY), hundred seed weight (HSDWT) and days to 50% flowering, evaluated across two locations in Norther Nigeria in year 2019

|  |  | Traits | Loc | Loc(Rep) | Loc(Rep (Block) | Geno | Geno*Loc | EMS | LEE | Mean | Min | Max | SEM | CV% |
| --- | --- | --- | --- | --- | --- | --- | --- | --- | --- | --- | --- | --- | --- | --- |
| Prelim1 | DF |  | 1 | 2 | 28 | 79 | 79 | 130 | 130 |  |  |  |  |  |
|  | MS | GY | 24985848*** | 98150ns | 94356** | 213,765*** | 135,067*** | 48,836 | 36,234 | 1,2378 | 514 | 1,819 | 135 | 15.4 |
|  |  | HSDWT | 34.40* | 0.80ns | 1.40ns | 20.8*** | 2.71*** | 2.2 | 1.2 | 15.7 | 7.3 | 21.4 | 0.8 | 7.0 |
|  |  | DT50F | 1162.8* | 31.10** | 4.00ns | 31.1*** | 13.1*** | 4.4 | 3.3 | 47.9 | 41.3 | 52.5 | 1.3 | 3.8 |
| Prelim2 | DF |  | 1 | 2 | 20 | 77 | 77 | 134 | 134 |  |  |  |  |  |
|  | MS | GY | 24847459* | 418038** | 57434ns | 213,744*** | 89,578*** | 53,071 | 31,752 | 1,094 | 1667 | 1,791 | 126 | 16.3 |
|  |  | HSDWT | 1.676ns | 17.34** | 2.09ns | 24.3*** | 1.88*** | 1.6 | 0.8 | 16.0 | 6.5 | 22.4 | 0.6 | 5.6 |
|  |  | DT50F | 729.30** | 4.50ns | 2.70ns | 22.0*** | 7.7*** | 4.6 | 2.7 | 48.8 | 41.8 | 53.8 | 1.2 | 3.4 |
| Prelim3 | DF |  | 1 | 2 | 28 | 71 | 71 | 114 | 114 |  |  |  |  |  |
|  | MS | GY | 2119562ns | 543380* | 110972** | 143,356*** | 109,007*** | 55,156 | 33,748 | 1,027.0 | 553 | 1,611 | 129.9 | 17.8 |
|  |  | HSDWT | 210.13* | 8.26* | 1.66ns | 18.2*** | 2.8*** | 1.2 | 0.9 | 15.9 | 12.0 | 22.0 | 0.7 | 5.9 |
|  |  | DT50F | 366.80ns | 30.70** | 4.40* | 11.9*** | 15.2*** | 2.9 | 2.5 | 49.9 | 46.3 | 54.0 | 1.1 | 3.2 |
| Prelim5 | DF |  | 1 | 2 | 28 | 79 | 79 | 130 | 130 |  |  |  |  |  |
|  | MS | GY | 946243ns | 486689* | 109702ns | 397,585*** | 292,284*** | 97,108 | 71,972 | 1,271 | 128 | 2,028 | 189.7 | 21.1 |
|  |  | HSDWT | 1.69ns | 20.35*** | 1.91ns | 20.3*** | 4.3*** | 3.1 | 1.8 | 15.9 | 8.3 | 21.3 | 0.9 | 8.4 |
|  |  | DT50F | 1101** | 3.60ns | 6.80ns | 36.3*** | 18.7*** | 5.7 | 4.5 | 48.5 | 41.8 | 58.3 | 1.5 | 4.4 |
| Prelim7 | DF |  | 1 | 2 | 20 | 77 | 77 | 134 | 134 |  |  |  |  |  |
|  | MS | GY | 517257ns | 475035* | 103274* | 309,134*** | 155,992*** | 61,853 | 45,481 | 1,098 | 338 | 1,862 | 150.8 | 19.4 |
|  |  | HSDWT | 99.81ns | 14.71*** | 1.42ns | 23.1*** | 3.4*** | 2.1 | 1.4 | 16.2 | 7.1 | 21.3 | 0.8 | 7.1 |
|  |  | DT50F | 1174* | 31.70** | 5.00ns | 17.4*** | 12.4*** | 4.5 | 3.1 | 46.8 | 41.3 | 52.0 | 1.3 | 3.8 |
| Prelim8 | DF |  | 1 | 2 | 28 | 71 | 71 | 114 | 114 |  |  |  |  |  |
|  | MS | GY | 11021273* | 481152* | 126568ns | 286,337*** | 179,715*** | 101,088 | 55,511 | 1,179 | 369 | 1,695. | 166.6 | 19.9 |
|  |  | HSDWT | 139.09ns | 22.26** | 3.26* | 17.0*** | 3.70*** | 1.9 | 1.4 | 16.8 | 13.7 | 24.0 | 0.8 | 7.0 |
|  |  | DT50F | 5547** | 45.00*** | 4.00ns | 40.0*** | 32.00*** | 7.0 | 5.6 | 54.2 | 45.0 | 60.5 | 1.7 | 4.4 |
| Prelim10 | DF |  | 1 | 2 | 14 | 63 | 63 | 98 | 98 |  |  |  |  |  |
|  | MS | GY | 10841803ns | 844077** | 127779ns | 167,201*** | 115,089*** | 73,121 | 27,707 | 1,237 | 798 | 1,715 | 117.7 | 13.5 |
|  |  | HSDWT | 238* | 4.22ns | 2.48* | 21.3*** | 2.6*** | 1.1 | 0.9 | 13.9 | 9.5 | 22.1 | 0.7 | 6.9 |
|  |  | DT50F | 479** | 0.30ns | 3.10ns | 18.1*** | 3.3*** | 3.5 | 1.4 | 46.5 | 41.5 | 50.0 | 0.8 | 2.6 |
| Prelim11 | DF |  | 1 | 2 | 32 | 89 | 89 | 146 | 146 |  |  |  |  |  |
|  | MS | GY | 5384045** | 64491ns | 227748*** | 184,667*** | 126,074*** | 45,953 | 32,513 | 804 | 418 | 1,404 | 127.5 | 22.4 |
|  |  | HSDWT | 857** | 5.20ns | 3.40* | 31.2*** | 3.4*** | 2.2 | 1.4 | 15.7 | 9.9 | 23.4 | 0.8 | 7.6 |
|  |  | DT50F | 1084** | 5.90ns | 2.80* | 14.6*** | 4.6*** | 1.8 | 1.4 | 45.5 | 39.8 | 51.0 | 0.8 | 2.5 |
| Denominator F-test | | | Loc(Rep) | Loc(Rep (Block) | EMS | LEE | LEE |  |  |  |  |  |  |  |

Geno= Genotype; Loc=Location; Rep=Replication; EMS=Error mean square; LEE =lattice effective error; Min=Minimum; Max=Maximum; SEM=Standard error of the mean; CV=Coefficient of variation; Df=Degrees of freedom; MS=Mean square; GY= Grain yield; HSDWT=Hundred seed weight; DT50F=Days to 50% flowering; the symbols; *,**, and *** represents the probability at 0.05,0.01 and 0.001 respectively.

**Genetic worth of multiple sets of cowpea breeding lines destined for advanced yield testing**

Patrick Obia Ongom^1, #^, Christian Fatokun^2^, Abou Togola^1^, Oyebode Gideon Oluwaseye^1^, Ahmad Mansur^1^, Ishaya Daniel Jockson^1^, Garba Bala^1^, Ousmane Boukar^1^

^1^International Institute of Tropical Agriculture (IITA), Kano, Nigeria

^2^International Institute of Tropical Agriculture (IITA), Ibadan, Nigeria

^#^correspondence;

E-mail: P.Ongom@cgiar.org

ORCID: https://orcid.org/0000-0002-5303-3602

Address: IITA Kano station, PMB 3112, Kano, Nigeria
