# Supplementary material for: Genetic worth of multiple sets of cowpea breeding lines destined for advanced yield testing
Source: Euphytica. 2021 Jan 29;217(2):30. doi: 10.1007/s10681-020-02763-y (PMC7846544; doi:10.1007/s10681-020-02763-y)
Supplement: Supplementary file 3 — Supplementary file3 (DOC 1,937 kb) [file 10681_2020_2763_MOESM3_ESM.doc]

**Supplementary Figure 2** PCA Clustering of genotypes within breeding sets. PCA showing the diversity within each of the eight breeding sets, each set is grouped by grain yield (GY), 100 seed weight (HSDWT) and days to 50% flowering (DT50FL). The circles within each PCA plot highlights genotypes within sets with high, intermediate and low values for GY, HSDWT and DT50FL. Genotypes within sets are named with codes; G1, G2, G3 and so on, with checks indicated as C1 and C2. The arrows pointing to the variables (GY, HDSWT and DT50FL) indicate the direction of traits contribution to variation explained by PC1 and PC2, which also corresponds to the depicted vertical and horozal orientations of the circles: Forexample; In prelim1, GY and HSDWT are associated with PC1 axis (hrizonatal direction and orientation) which account for 45.4% of variation while DT50 is associated with PC2 axis (veritcal direction/orientation) which expained the remaining variations.

**Supplementary Figure 2 (Continue)** PCA Clustering of genotypes within breeding sets . PCA showing the diversity within each of the eight breeding sets, each set is grouped by grain yield (GY, kg/ha), 100 seed weight (HSDWT, g) and days to 50% flowering (DT50FL). The circles within each PCA plot highlights genotypes within sets with high, intermediate and low values for GY, HSDWT and DT50FL. Genotypes within sets are named with codes; G1, G2, G3 and so on, with checks indicated as C1 and C2. The arrows pointing to the variables (GY, HDSWT and DT50FL) indicate the direction of traits contribution to variation explained by PC1 and PC2, which also corresponds to the depicted vertical and horozal orientations of the circles: Forexample; In prelim1, GY and HSDWT are associated with PC1 axis (hrizonatal direction and orientation) which account for 45.4% of variation while DT50 is associated with PC2 axis (veritcal direction/orientation) which expained the remaining variations.

**Genetic worth of multiple sets of cowpea breeding lines destined for advanced yield testing**

Patrick Obia Ongom^1, #^, Christian Fatokun^2^, Abou Togola^1^, Oyebode Gideon Oluwaseye^1^, Ahmad Mansur^1^, Ishaya Daniel Jockson^1^, Garba Bala^1^, Boukar Ousmane^1^

^1^International Institute of Tropical Agriculture (IITA), Kano, Nigeria

^2^International Institute of Tropical Agriculture (IITA), Ibadan, Nigeria

^#^correspondence;

E-mail: P.Ongom@cgiar.org

ORCID: https://orcid.org/0000-0002-5303-3602

Address: IITA Kano station, PMB 3112, Kano, Nigeria
