# Supplementary material for: Genetic worth of multiple sets of cowpea breeding lines destined for advanced yield testing
Source: Euphytica. 2021 Jan 29;217(2):30. doi: 10.1007/s10681-020-02763-y (PMC7846544; doi:10.1007/s10681-020-02763-y)
Supplement: Supplementary file 4 — Supplementary file4 (DOCX 423 kb) [file 10681_2020_2763_MOESM4_ESM.docx]

 **Supplementary Figure 1** Traits distributions per breeding set. Each Histogram is presented together with a corresponding boxplot comparing performance between two test locations. **a** distribution for grain yield (GY). **b** 100 Seed weight (HSDWT). **c** number of days to 50% flowering (D50FL). The eight sets of breeding materials are named: Prelim1, Prelim2, Prelim3, Prelim4, Prelim5, Prelim7, Prelim8, Prelim10, Prelim11 and were evaluated in 2019 at two locations (Minjibir and Shika) in Northern Nigeria.

**Genetic worth of multiple sets of cowpea breeding lines destined for advanced yield testing**

Patrick Obia Ongom^1, #^, Christian Fatokun^2^, Abou Togola^1^, Oyebode Gideon Oluwaseye^1^, Ahmad Mansur^1^, Ishaya Daniel Jockson^1^, Garba Bala^1^, Boukar Ousmane^1^

^1^International Institute of Tropical Agriculture (IITA), Kano, Nigeria

^2^International Institute of Tropical Agriculture (IITA), Ibadan, Nigeria

^#^correspondence;

E-mail: P.Ongom@cgiar.org

ORCID: https://orcid.org/0000-0002-5303-3602

Address: IITA Kano station, PMB 3112, Kano, Nigeria
