# Supplementary material for: Genetic worth of multiple sets of cowpea breeding lines destined for advanced yield testing
Source: Euphytica. 2021 Jan 29;217(2):30. doi: 10.1007/s10681-020-02763-y (PMC7846544; doi:10.1007/s10681-020-02763-y)
Supplement: Supplementary file 6 — Supplementary file6 (DOCX 16 kb) [file 10681_2020_2763_MOESM6_ESM.docx]

**Supplementary Table 1** Overall means for grain yield, hundred seed weight and days to 50% flowering of eight cowpea breeding sets previously created based on maturity duration, evaluated across two locations in Northern Nigeria during the year 2019.

|  |  | GY (Kg/ha) | | | HSDWT (g) | | | DT50FL (days) | | |
| --- | --- | --- | --- | --- | --- | --- | --- | --- | --- | --- |
| Set | Size | Minjibir | Shika | Mean | Minjibir | Shika | Mean | Minjibir | Shika | Mean |
| Prelim1 | 80 | 1,197.90 | 1,277.52 | 1,237.71 | 15.41 | 16.07 | 15.74 | 46.06 | 49.87 | 47.97 |
| Prelim2 | 78 | 1,006.91 | 1,174.18 | 1,090.55 | 16.12 | 15.97 | 16.05 | 47.24 | 50.28 | 48.76 |
| Prelim3 | 72 | 1,139.72 | 931.05 | 1,035.39 | 16.72 | 15.01 | 15.87 | 51.12 | 48.86 | 49.99 |
| Prelim5 | 80 | 1,202.00 | 1,332.28 | 1,267.14 | 15.97 | 16.06 | 16.02 | 46.64 | 50.38 | 48.51 |
| Prelim7 | 78 | 1,043.51 | 1,148.90 | 1,096.21 | 16.76 | 15.61 | 16.19 | 44.83 | 48.69 | 46.76 |
| Prelim8 | 72 | 1,379.46 | 970.33 | 1,174.90 | 17.51 | 16.1 | 16.81 | 58.61 | 49.84 | 54.23 |
| Prelim10 | 64 | 1,032.91 | 1,457.64 | 1,245.28 | 14.92 | 12.98 | 13.95 | 45.09 | 47.82 | 46.46 |
| Prelim11 | 90 | 687.97 | 890.3 | 789.14 | 17.23 | 14.06 | 15.65 | 43.82 | 47.27 | 45.55 |

Minjibir and Shika are the two locations at which the sets were evaluated.

**Genetic worth of multiple sets of cowpea breeding lines destined for advanced yield testing**

Patrick Obia Ongom^1, #^, Christian Fatokun^2^, Abou Togola^1^, Oyebode Gideon Oluwaseye^1^, Ahmad Mansur^1^, Ishaya Daniel Jockson^1^, Garba Bala^1^, Boukar Ousmane^1^

^1^International Institute of Tropical Agriculture (IITA), Kano, Nigeria

^2^International Institute of Tropical Agriculture (IITA), Ibadan, Nigeria

^#^correspondence;

E-mail: P.Ongom@cgiar.org

ORCID: https://orcid.org/0000-0002-5303-3602

Address: IITA Kano station, PMB 3112, Kano, Nigeria
